# Neutrophil-to-Lymphocyte Ratio as a Biomarker for Motor Subtypes in Idiopathic Parkinson’s Disease

**DOI:** 10.7759/cureus.77440

**Published:** 2025-01-14

**Authors:** Rashid Awan, Okasha Tahir, Shahzad Noor Ul Hadi, Wajeeh Ur Rehman, Fahad Asim

**Affiliations:** 1 Medicine, Chiniot General Hospital, Karachi, PAK; 2 Medicine, Bacha Khan Medical College, Mardan, PAK; 3 Medicine, Lady Reading Hospital, Peshawar, PAK; 4 Internal Medicine, Saidu Group of Teaching Hospitals, Swat, PAK; 5 Pharmacology and Therapeutics, Faculty of Pharmacy, The University of Lahore, Lahore, PAK

**Keywords:** client satisfaction, neutrophil count, neutrophil-to-lymphocyte ratio (nlr), parkinson's disease, patient satisfaction

## Abstract

Background: Parkinson’s disease (PD) is one of the most common neurodegenerative disorders, primarily affecting movement and motor function. Described by the symptoms, including tremors, rigidity, bradykinesia, and postural instability, PD presents clinical heterogeneity in patients, which complicates diagnostic and therapeutic interventions.

Objective: The study aimed to find the neutrophil-to-lymphocyte ratio (NLR) as a biomarker for motor subtypes in idiopathic PD.

Materials and methods: This prospective observational study was conducted at Chiniot General Hospital Karachi, Pakistan, from January 2023 to January 2024. Data were collected from 55 patients suffering from idiopathic PD. Demographic and clinical data were collected for each patient, including age, sex, disease duration, and medications used for PD management.

Results: There was no significant difference between the tremor-dominant (TD) and postural instability and gait difficulty (PIGD) subtypes in terms of age (p = 0.45), sex distribution (p = 0.62), or disease duration (p = 0.68). However, the Hoehn and Yahr scale, which measures disease severity, was significantly higher in the PIGD subtype (2.9 ± 0.6) compared to the TD subtype (2.4 ± 0.5) with a p-value of 0.03, indicating greater disease severity in the PIGD group. The results indicate that the NLR was significantly higher in the PIGD subtype (3.17 ± 0.89) than in the TD subtype (2.41 ± 0.72), with a p-value of 0.01. This suggests a stronger inflammatory response in the PIGD subtype, which could be linked to greater disease severity in these patients.

Conclusion: The NLR can be a potential biomarker for distinguishing motor subtypes in idiopathic PD.

## Introduction

Parkinson’s disease (PD) is one of the most common neurodegenerative disorders, primarily affecting movement and motor function. Described by the symptoms, including tremors, rigidity, bradykinesia, and postural instability, PD presents clinical heterogeneity in patients, which complicates diagnostic and therapeutic interventions. The precise etiology of PD has not been identified, although it is thought to be brought about by the degeneration of dopaminergic neurons in the substantia nigra [1]. The primary focus is clinical variability in the initial presentation and motor subtypes that require biomarkers for early diagnosis, prognosis, and related treatment options [2]. Of such potential novel biomarkers is the neutrophil-to-lymphocyte ratio (NLR), which reflects a mark of the level of inflammation. It has been proposed that inflammation, as an integral component of systemic inflammation, plays some role in PD disease development [3].

Persons with chronic inflammation in the CNS systemically contribute to further neuronal damage and progression. Lymphocytes are immune cells, and neutrophils can be viewed in that context. Their ratio is NLR, which reflects the body’s inflammatory levels [4]. Neutrophils are linked to acute inflammation, while lymphocyte results indicate an adaptive immune response. An increased NLR indicates that inflammation plays a role in tissue and organs. In the last few years, the inflammation aspect of PD has been given much focus and attention. Such microglial activation, pro-inflammatory cytokines, and oxidative stress have all been observed to contribute to the death of dopaminergic neurons [5].

The NLR has evolved as this study identifies it as a potential marker of cellular inflammation in PD that is simple, affordable, and can be implemented in clinics. Some works have shown a correlation between NLR and PD: there is evidence that high NLR is connected with the severity and worsening of the disease [6]. Unfortunately, there is a poor evidential record to support the prospect of using NLR as an index of motor subtypes in PD. In contrast, PD is most commonly distinguished according to the clinical presentation of the subtypes of motor dysfunction. Some of the more common subtypes are the tremor-dominant (TD) subtype, which results in mostly tremors, and the postural instability and gait difficulty (PIGD) subtype: patients suffering from this type of PD have severe balance and movement abnormalities [7]. These are linked with various disease patterns, prognoses, and treatment responses. Elucidating the relationship between NLR and these motor subtypes could provide further insight into the inflammation implicated in PD’s clinical variability [8].

Although emerging literature reports on inflammation in PD and, in particular, the link between NLR and PD, few studies have attempted to investigate the association between NLR and the specific motor subphenotypes of idiopathic PD. While several research works have examined the overall disease course or broad indices of inflammation, few have demonstrated how exactly NLR may differ between the TD and PIGD types of PD. However, to our knowledge, no systematic attempts have been made to examine NLR as a specific biomarker for dissecting the motor subtypes of idiopathic PD, which would facilitate a finer understanding of the inflammatory aspect of the disease.

The study's main objective is to find the NLR as a biomarker for motor subtypes in idiopathic PD.

## Materials and methods

This prospective observational study was conducted at Chiniot General Hospital Karachi, Pakistan, from January 2023 to January 2024. The Institutional Review Board of Chiniot General Hospital issued approval 011-022-23. Data were collected from 55 patients suffering from idiopathic PD using convenience sampling. The sample size was calculated using an OpenEpi (Dean AG, Sullivan KM, Soe MM. OpenEpi: Open Source Epidemiologic Statistics for Public Health, www.OpenEpi.com) calculator based on the formula for estimating proportions for a population with a confidence level of 95% and a margin of error of 5% [9].

Inclusion and exclusion criteria

Patients with a confirmed diagnosis of idiopathic PD and aged between 40 and 80 years with no history of significant comorbidities, including autoimmune diseases, cancer, or chronic infections, which could influence systemic inflammatory markers like NLR, were included in the study. Patients who were not on anti-inflammatory medications or immunosuppressants were also included.

Patients with secondary PD, atypical PD syndromes, or any history of acute infections or surgeries within three months prior to the study were excluded to avoid confounding effects on inflammatory markers.

Data collection

The study participants were classified into two major motor subtypes: one is the TD subtype, and the second is the PIGD subtype. The classification into motor subtypes was based on the Movement Disorder Society Unified Parkinson’s Disease Rating Scale (MDS-UPDRS) [10], particularly focusing on the motor examination section (Part III). Patients were assessed using the scale's TD- and PIGD-related items to assign them to the respective motor subtype. Upon recruitment, detailed demographic and clinical data were collected for each patient, including age, sex, disease duration, and medications used for PD management. A comprehensive neurological examination was conducted to assess motor symptoms, severity of disease, and stage using the Hoehn and Yahr scale. A complete blood count (CBC) was performed on a venous blood sample obtained from each participant after an overnight fast to evaluate systemic inflammation. The NLR was calculated by dividing the absolute neutrophil count by the absolute lymphocyte count, both derived from the CBC results.

Statistical analysis

Data were analyzed using SPSS Statistics version 29 (IBM Corp. Released 2019. IBM SPSS Statistics for Windows, Version 26.0. Armonk, NY: IBM Corp.). Descriptive statistics were used to summarize the demographic and clinical characteristics of the study population. Continuous variables were presented as means ± standard deviations, while categorical variables were expressed as frequencies and percentages. Chi-square and t-tests were used to analyze associations between primary and secondary outcomes, with a p-value of <0.05 considered statistically significant.

## Results

Data were collected from 55 patients, and there was no significant difference between the TD and PIGD subtypes in terms of age (p = 0.45), sex distribution (p = 0.62), or disease duration (p = 0.68). However, the Hoehn and Yahr scale, which measures disease severity, was significantly higher in the PIGD subtype (2.9 ± 0.6) compared to the TD subtype (2.4 ± 0.5) with a p-value of 0.03, indicating greater disease severity in the PIGD group (Table 1).

**Table 1:** Demographic values TD: Parkinson’s disease, PIGD: postural instability and gait difficulty

Characteristic	TD subtype (n=32)	PIGD subtype (n=23)	p-value
Age (years)	64.8 ± 7.9	66.1 ± 8.3	0.45
Sex (male %)	59.4%	52.2%	0.62
Disease duration (years)	7.1 ± 3.5	7.6 ± 3.7	0.68
Hoehn and Yahr scale	2.4 ± 0.5	2.9 ± 0.6	0.03

The study's motor subtype distribution shows that most patients fell into the PIGD subtype, with 25 patients (45%). The TD subtype accounted for 20 patients (36%), while the mixed subtype was the least common, comprising 10 patients (18%). This distribution highlights the predominance of the PIGD subtype in the study population. The NLR was significantly higher in the PIGD subtype (3.17 ± 0.89) compared to the TD subtype (2.41 ± 0.72), with a p-value of 0.01 (Table 2).

**Table 2:** NLR values NLR: neutrophil-to-lymphocyte ratio, TD: Parkinson’s disease, PIGD: postural instability and gait difficulty

Characteristic	TD subtype (n=32)	PIGD subtype (n=23)	p-value
NLR	2.41 ± 0.72	3.17 ± 0.89	0.01

NLR was significantly correlated with the MDS-UPDRS score (r = 0.43, p = 0.02) and the Hoehn and Yahr scale (r = 0.39, p = 0.03). These findings suggest that higher NLR levels are associated with greater motor symptom severity and more advanced disease stages in PD (Table 3).

**Table 3:** Correlation between NLR and disease duration NLR: neutrophil-to-lymphocyte ratio, MDS-UPDRS: Movement Disorder Society Unified Parkinson’s Disease Rating Scale

Correlation	r-value	p-value
NLR vs. MDS-UPDRS	0.43	0.02
NLR vs. Hoehn and Yahr	0.39	0.03

## Discussion

The present research aimed to assess the NLR as a biomarker to differentiate motor subtypes in individuals with idiopathic PD. Specifically, our study proposes that the NLR is higher in the PIGD subtype, confirming the hypothesis that systemic inflammation plays a more significant role. We also documented a direct relationship between NLR and disease severity, suggesting that NLR could be a biomarker for disease progression in PD [11]. However, there has been a relative neglect of the general inflammation, which is easier to evaluate in the peripheral blood markers than the core concept. By this view, NLR may be more ecologically valid than other markers of peripheral immune activation that have previously been linked to CNS pathology in PD. NLR is simple and more readily available than other markers of systemic inflammation. Our findings support the evidence of higher inflammatory levels in the PIGD subtype rather than the TD subtype seen in earlier studies. PIGD subtype is observed in patients revealing more severe motor abnormalities, rapid clinical progression, and fewer favorable outcomes [12]. The higher NLR levels observed in the PIGD group may indicate more severe systemic inflammation in these patients, possibly associated with the worse prognosis in this cohort. These observations correspond to the increasing literature on inflammation as a causative factor in neurodegeneration and motor disability in PD [13]. Another important result of this study is the difference in NLR between the TD and PIGD subtypes. Since the PIGD subtype is related to a shorter survival time, it might be beneficial to accurately diagnose clients with this subtype to provide more personalized treatment approaches [14]. This shows the possibility of the clinical application of NLR for assessing the severity of the disease and making decisions on further management. As such, the following are the health implications of the findings of this study. First, as shown, NLR determination is very simple, does not require considerable financial investment, and thus could easily be integrated into the regular clinical practice. As NLR can be obtained from a normal CBC, it is a non-invasive and highly accessible biomarker for PD patients [15]. Second, NLR could be utilized as a supplementary diagnostic aid for subtype differentiation in PD patients, increasing the reliability of nearly accurate decisions regarding treatment choices. The overall severity of the disease was significantly different according to the Hoehn and Yahr scale [10] between the TD and the PIGD subtypes. The PIGD group had a statistically significant high mean score of 2.9 ± 0.6 compared to the TD group (2.4 ± 0.5, p = 0.03). This suggests that the PIGD subtype patients are rated to have greater severity of illness in general, which is typical in PD, where PIGD are signs of advanced disease. This finding is also comparable to prior studies that show that the PIGD subtype of patients displays higher levels of functional disability and a more rapid disease progression than patients in the TD subtype. The subsequent analysis of the PIGD subtype, characterized by higher disease severity, also strengthens the assumption that different motor signs may reflect various disease courses [16].

Limitations of the study

The following limitations are worth revealing regarding this study: First, it involved a small sample size of only 55 patients, and second, the number of patients with a history of alcohol use was also small, with only 14 of our sample patients having a history of alcohol use. We found gender differences in the NLR levels across the PD subtypes. This suggests that future work with larger sample sizes should replicate the current findings examining the relationship because NLR can help subtype PD for a specific gender. Second, in this study, we only recruited patients with idiopathic PD; therefore, it is uncertain whether NLR could be applied to atypical PD patients.

## Conclusions

The NLR can be a potential biomarker for distinguishing motor subtypes in idiopathic PD. Higher NLR levels were associated with the more severe PIGD subtype, as well as greater overall disease severity. These findings highlight the role of systemic inflammation in PD progression and support the use of NLR in clinical practice for disease monitoring and subtype differentiation.
